# Does group cohesion foster self-directed learning for medical students? A longitudinal study

**DOI:** 10.1186/s12909-020-1962-7

**Published:** 2020-02-21

**Authors:** Soyun Kim, Eunbae B. Yang

**Affiliations:** Department of Medical Education, Yonsei University College of Medicine, # 421, 50-1 Yonsei-ro, Seodaemun-gu, Seoul, 03722 South Korea

**Keywords:** Self-directed learning readiness, Group cohesion, Medical students, Criterion-referenced grading system

## Abstract

**Background:**

The importance of self-directed learning (SDL) and collaborative learning has been emphasized in medical education. This study examined if there were changes in the pattern of SDL and group cohesion from the time of admission to medical school under the criterion-referenced grading system, increased group activities, and interaction of medical education curriculum. Second, it was examined whether group cohesion influences self-directed learning.

**Methods:**

The participants were 106 medical students (71 males, 35 females) who enrolled in Yonsei University College of Medicine in Seoul, South Korea in March 2014. They were asked to complete a Korean version of the self-directed learning readiness scale (SDLRS) and group cohesion scale (GCS) at the end of each semester for three years. A repeated measures ANOVA and a correlation and regression analysis were conducted.

**Results:**

All the participants completed the questionnaires. There were differences in the SDLRS scores over the three years. A significant increase was observed one year after admission followed by stable scores until the third year. There was a significant increase in GCS scores as students progressed through medical school years. Positive relationships were found between SDLRS and GCS scores, and the regression model predicted 32% variance.

**Conclusions:**

SDLRS and GCS increased as medical school years progressed. In addition, GCS is a significant factor in fostering SDLRS. Medical schools should develop various curriculum activities that enhance group cohesion among medical students, which would in turn promote SDL.

## Background

Lifelong learning is one of the most emphasized components of being a successful physician. As a promising methodology for lifelong learning, SDL has been recommended in medicine [1–3] and has increasingly been emphasized as an important principle in medical education. Several definitions of SDL have been suggested in the literature [4–8]. The commonly accepted definition of SDL is that of Knowles [4], who defined it as a learning process in which individuals take the initiative in diagnosing their learning needs, formulating goals, identifying human and material sources, selecting and implementing strategies, and evaluating learning outcomes. The scholar role of the ‘CanMEDS 2015 Physician Competency Framework’ emphasized the ‘lifelong learner’ component by describing three enabling competencies: 1) the need for a personal learning plan, 2) the use of data from a variety of sources to guide learning, and 3) the importance of collaborative learning [9]. The American Board of Medical Specialties established ‘Maintenance of Certification’, including lifelong learning as one of its components and self-directed learning (SDL) as a characteristic to be encouraged by medical education providers [10]. Researchers asserted that ‘SDL represents the ultimate state of learner autonomy: the learner exercises control over and major responsibility for choosing both the goals and the means of the learning’ [5]. Several researchers have characterized and defined self-directed learners as independent, autonomous, and having self-control [4, 11].

Recently, studies which have investigated factors that influence SDL have shown that SDL does not work alone. Giddings [12] emphasized that SDL is not just an individual’s work. She noted that SDL is a dynamic concept that has functional relationships with several educational dimensions: a learner’s metacognitive behavior, the social context in which learning occurs, and the pedagogical dimension of the interaction between teaching and learning. Baskett [13] identified workplace factors that improve SDL and found that effective communication systems, an environment of trust and mutual respect, and collaboration among organization members were all important factors for enhancing SDL. Previous studies have shown that self-directed learning involves interaction with peers and teachers to exchange information [2, 14].

Intensive interaction with peers occurs in medical schools because classmates share similar class schedules. Peers will also have almost the same experience in classes and clinical clerkship for the entire duration of the program. Once admitted into the program, medical students spend most of their time with their peers, interacting over curricular and extracurricular activities. By engaging in diverse activities during their school years, medical students influence each other. Peers are significant in medical school life. Group cohesion is one of the most widely used constructs to measure relationships in clinical and empirical literature [14]. While there are several definitions of group cohesion, it is generally identified as a sense of bonding or working together towards common goals, mutual acceptance, and identification and affiliation with a group [14, 15].

Highly competitive environments and teacher-centered learning which leads to passive learning have been increasing concerns for medical students. In a traditional education paradigm, a multi-tiered norm-referenced grading system (e.g. A, B, C, F) that assesses the relative performance of individuals has been used. Several issues have been raised with this grading system, such as excessive competition with colleagues, distress, a decrease in extrinsic motivation, and passive learning. Yonsei University College of Medicine has adopted the educational philosophy that a change in grading systems will change students’ attitudes towards learning and the learning environment. In 2014, the institute changed the grading system from a multi-tiered norm-referenced grading system (e.g. A, B, C, F) to a two-tiered criterion-referenced grading system (e.g. pass, non-pass). Additionally, several courses were designed to encourage group activities by interacting with each other, such as small group lectures. Extracurricular activities such as learning communities were developed, aimed at giving students the opportunity for collaborative learning with peers, as well as self-directed and experiential learning. Previous studies have shown that pass and fail grading systems seem to reduce students’ stress and promote group cohesion [16]. White and Fanteone [17] reported that a pass/non-pass grading system promotes intrinsic motivation and self-regulated learning. Furthermore, cohesive groups seem to outperform non-cohesive groups, and have greater job and personal satisfaction, [18] while also having positive effects on an individual’s contribution to a group [19]. Our faculty expected that a change in the grading system and the design of several curricular and extracurricular activities would promote interaction among medical students, which would lead to self-directed learning.

The purpose of this study was to analyze the relationship between group cohesion and self-directed learning under the changed criterion-referenced grading system and curriculum, focusing on group activities and small group lectures. To achieve this, the study examined if there were changes in the pattern of SDL and group cohesion since the admission of students into the institution. It then examined whether group cohesion influences SDL. While previous studies have shown that there was a significant decrease in SDL after admission during medical school training [2, 20], this study hypothesized that there would be no decrease in SDL or group cohesion with school years. Group cohesion was expected to foster SDL.

## Methods

### Participants

The participants were 106 medical students (71 males, 35 females) who enrolled in Yonsei University College of Medicine in Seoul, South Korea in March 2014.

### Instruments

#### Self-directed learning readiness scale (SDLRS)

Although many instruments have been developed to assess SDL, there are few translated Korean versions. Existing studies initially stated that Kim and her colleagues translated Guglielmino’s Self-Directed Learning Readiness Scale [21] into Korean (i.e. SDLRS-K-91). Due to cultural differences, several items were revised by researchers later, and a new measure – SDLRS-K-96 – was developed for Korean primary school teachers [22]. Based on the SDLRS-K-96, Han developed a revised version of SDLRS for Korean college students. Initially, Han revised SDLRS-K-96 [23] and conducted factor analyses seven times, yielding a total of 23 items by deleting 35 out of 58 items. They consisted of seven constructs: love of learning, openness towards learning, self-perception, basic learning function and independence, acceptance of responsibility for learning, leadership and future directivity, and creativity and exploration [23]. A five-point Likert scale was used with text description and the following anchors: (1) strongly disagree; (2) disagree; (3) disagree or agree; (4) agree; and (5) strongly agree. We obtained permission for its use from the author.

#### Group cohesion scale (GCS)

Group cohesion has been studied in several disciplines over the past several decades. To assess group cohesion, GCS was used for this study [24]. Although it was originally designed for psychiatric inpatients, the authors suggested that it may be used in any type of group activity in which interaction is involved. After changing the grading system and part of the curriculum in our medical school, group activity was encouraged during regular classes with a form of team-based learning and extracurricular activities, such as the learning community designed to promote peer collaborative learning and work by interacting with each other.

Initially GCS was translated for the current study by researchers; one researcher (PhD in medical education) translated all items of GCS from English to Korean. After the initial translation process, four researchers (two PhDs in psychology, one PhD in education, and one psychiatrist) reviewed and revised the items. The translated Korean version was back-translated into English. The final version was revised until the researchers reached consensus. GCS comprises a seven-item, five-point, Likert-type scale ranging from (1) strongly disagree to (5) strongly agree.

### Data collection and statistical analysis

Prior to administration, the purpose of this study was explained by a researcher. Participants were told that the study was regular educational practice, held every year. Since our school had changed the grading system from norm-referenced to criterion-referenced since 2014, the changes needed to be monitored for the results to be reflected in educational management and policy. Students were also asked to read and sign an informed consent form, which explained that their names would be saved anonymously, data kept confidential, and that participation in this study was voluntary and they could decline to participate at any time. After signing the informed consent form, participants were given a set of questionnaires, followed by a demographic information form. The questionnaires were in paper form. The survey took approximately 30 min. The survey was administered at the end of every first semester for each year that the student was enrolled. This study falls in the category of Ethical approval exemption under Article 2 of the Enforcement Regulations of the Bioethics and Safety in the Health-Welfare Ministry in South Korea, where it is stated that a research is exempt if it involves only normal educational practices.

All collected data were kept confidential and anonymous. On both, the informed consent forms and the questionnaires, randomized numbers were assigned prior to the survey. Consent forms and questionnaires were saved and coded separately. Research assistants coded participants’ names on the consent forms, and the questionnaires were coded with randomized numbers. After collecting data for every year, the randomized numbers were matched to participants’ names that were linked with the data from the previous year.

All analyses were done using SPSS version 23 (IBM Corp., Armonk, NY), and data collected from the questionnaires were entered in the SPSS Statistics Editor. Some data were excluded from the data set under the following criteria: 1) standard deviations of the answers were 0, indicating that participants were giving the same rating for all questions or 2) not participating for all three years. All reverse-coded items were re-coded and then analyzed using appropriate descriptive analysis, including mean and standard deviation. Because the data met the normality assumption, a repeated measures ANOVA was conducted to compare means from three years for each SDLRS and GCS.

Based on the correlation and causal-comparative design [25], the correlation coefficient to quantify the strength of the relationship between SDR and GCS variables was calculated. Regression was used to find the presence of a linear relationship and, if there is any relationship, to obtain the coefficient of determination (R^2^) from the analysis. The statistical significance was set at a *p*-value of less than or equal to .05.

## Results

Initially, 106 students (71 males, 35 females) participated. Response rates for three years were 100%, and attrition rate was 11.7%. Twelve responses were excluded because they did not satisfy the criteria described in the methods section. As a result, responses from 94 participants (61 males, 33 females) were analyzed. Owing to a change in the number of responses from the sample size initially planned, we conducted a post-hoc power analysis with the program G*Power version 3.1. to find whether our design had enough power to detect effects of GCS on SDLRS with alpha = .05. The power to detect an effect in this study was determined to be 0.99 in the repeated measures design with effect size = 0.25 (i.e. a medium effect, Cohen’s, 1977), 0.90 in the correlation matrix (H1 = 0.3, two-tailed, alpha = .05), and 0.95 in the regression model (effect size = 0.15, alpha = .05).

### SDLRS

Cronbach’s alpha reliability scores for each year (2014, 2015, 2016) were .782, .808, and .851 respectively, in the current study. The first analysis involved the SDLRS scores as a function of three years (Table 1). A one-way repeated measures ANOVA on the SDLRS mean indicated that there was a significant effect of years, *F* (2,182) = 3.212, MS*e* = .080, *p* < .05. The Bonferroni post-hoc test showed that the SDLRS score in the second year was the highest, followed by the third year with no significant difference between the two, while the first year was rated the lowest. To examine the effect of gender, a 3 (year: 1st vs. 2nd vs. 3rd) x 2 (gender: male vs. female) mixed-model ANOVA revealed that there was a significant main effect of year, *F* (2,180) = 3.387, MS*e* = .081, *p* < .05, but no effect of gender, *F* (1, 90) = .002, MS*e* = .324, *p* > .05, nor was there any interaction effect between year and gender, *F* (2, 180) = .307, MS*e* = .081, *p* > .05. Gender is a between-subject factor, and year is a within-subject factor.

**Table 1:** Mean and standard deviation of SDLRS scores by year and gender

Year	Grade	Overall (*n* = 94)	Male (*n* = 61)	Female (*n* = 33)
1st year (at admission)	1	3.68(.4)	3.68(.4)	3.68(.3)
2nd year	2	3.78(.4)	3.77(.4)	3.81(.4)
3rd year	3	3.71(.4)	3.72(.5)	3.69(.3)

A second main analysis specifically examined the seven subdomains of SDLRS scores and whether the subdomains changed over the three years (Table 2). Seven one-way repeated measures ANOVA were conducted for each subdomain of SDLRS, for which four subdomains indicated significant differences. ‘Openness to learning’, *F* (2, 186) = 4.535, MS*e* = .159, *p* < .05: the Bonferroni post-hoc test showed that the score in the second year was the highest and was significantly higher than the first and third years, with no significant difference between these two; 2) ‘Basic learning function and independence’ showed *F* (2, 184) = 3.139, MS*e* = .193, *p* < .05: the Bonferroni post-hoc test showed that the score in the second year was the highest and significantly different from the first year; no significant differences were found between other comparisons; 3) ‘Self-perception’, *F* (2, 186) = 5.617, MS*e* = .282, *p* < .01: the second year was the highest and significantly different from the third year scores, which were the lowest; 4) ‘Love of learning’, *F* (2, 184) = 6.156, MS*e* = .141, *p* < .01: the Bonferroni post-hoc test showed that the score in the third year was the highest and significantly different from the score in the first year; there was no difference between the second and third years, nor between the first and second.

**Table 2:** Mean and standard deviation of subdomains of SDLRS in descending order

Subdomains	Overall (*n* = 94)	Years		
		1st year (at admission)	2nd year	3rd year
Creativity and exploration	3.99(.4)	4.00(.6)	4.03(.6)	3.94(.6)
Openness for learning*	3.87(.5)	3.82(.5)	3.97(.5)	3.82(.5)
Acceptance of responsibility for learning	3.83(.6)	3.77(.6)	3.88(.6)	3.84(.6)
Basic learning function and independence*	3.77(.6)	3.68(.6)	3.85(.6)	3.77(.6)
Self-perception*	3.70(.7)	3.74(.6)	3.81(.7)	3.56(.8)
Leadership and future directivity	3.65(.6)	3.63(.6)	3.68(.6)	3.64(.6)
Love of learning*	3.47(.5)	3.38(.4)	3.48(.5)	3.56(.5)

* *p* < .05: significant difference as function of years

### GCS

To examine factor structure, a factor analysis was conducted using the Kaiser-Meyer-Olkin measure of sampling adequacy (KMO-test), which came out at .867. This indicates that the sample size was adequate; the Bartlett test was .000 suggesting no multicollinearity. Similar to the previous study [24], the results of factor analysis showed that only one factor was extracted. Cronbach’s alpha reliability scores for each year (2014, 2015, and 2016) were .770, .884, and .920, respectively.

A repeated measures ANOVA was conducted and rendered for scores as a function of each year, *F* (2,182) = 10.192, MS*e* = .167, *p* < .01 (Table 3). An additional Bonferroni post-hoc test showed that the GCS scores in the third year were higher than those in the second year without significant differences between the two, and those in the first year were the lowest, *p* < .01.

**Table 3:** Mean and standard deviation for GCS scores by year and gender

Year	Overall (*n* = 94)	Male (*n* = 61)	Female (*n* = 33)
1st year (at admission)	3.73 (.4)	3.75 (.4)	3.71 (.5)
2nd year	3.90 (.5)	3.90 (.5)	3.91 (.6)
3rd year	4.00 (.6)	4.00 (.7)	4.01 (.5)

A 3 (year) x 2 (gender: male vs. female) mixed-model ANOVA revealed that there was a significant main effect for year, *F (2,182)* = 5.244, MS*e* = 9.081, *p* < .01 but no significant effect for gender on interaction (*p* > .05). Gender is a between-subject factor, and grade is a within-subject factor.

### SDLRS and GCS

To examine the relationship between SDLRS and GCS, correlation and regression analysis were conducted. The correlation of overall mean scores between SDLRS and GCS scores accumulated for the three years showed a significant positive relationship (*r* = .57, *p* < .001). The correlation between the seven subdomains of SDLRS and GCS scores showed that all subdomains of SDLRS scores had significant positive relationships with GCS mean scores (*p* < .01). To examine the pattern for each year, correlation between the mean scores of SDLRS and GCS was conducted. There were significant positive relationships (*p* < .001), as shown in Table 4, which indicates that the higher the GCS mean scores, the higher the SDLRS mean scores in a given year.

**Table 4:** Correlation between SDLRS and GCS scores for three years

		SDLRS		GCS		
	Year	2015	2016	2014	2015	2016
SDLRS	2014	.62**	.37**	.41**	.31**	.19
	2015		.53**	.32**	.64**	.30**
	2016			.25**	.44**	.63**
GCS	2014				.47**	.43**
	2015					.44**

**p < .01

A linear regression analysis was conducted to ascertain the extent to which GCS scores can predict SDLRS scores. The model was a good fit for the data (*F* = 131.363, *p* < .001). The regression model predicted 32% variance. Additionally, a linear regression analysis was carried out for each subdomain of SDLRS with GCS scores (Table 5). The variance of the subdomain ‘acceptance of responsibility for learning’ was explained upto 25.8% by GCS scores; that of ‘creativity and exploration’ by 20.7%, and ‘love of learning’ by 19.6%. On the other hand, the variance of ‘leadership and future directivity’ and ‘self-perception’ were explained 9.3 and 9.5% by a GCS score, respectively.

**Table 5:** Regression analysis of each subdomain of SDLRS and overall GCS scores

	B	Standard error	Standardized coefficient	*F*	*p*
			beta		
(constant)					
Love of learning	.386	.047	.443	67.565	.000
Openness for learning	.388	.054	.393	50.642	.000
Self-perception	.386	.072	.308	29.095	.000
Basic learning function and independence	.442	.060	.406	54.630	.000
Acceptance of responsibility for learning	.528	.054	.508	96.832	.000
Leadership and future directivity	.327	.061	.305	28.434	.000
Creativity and exploration	.493	.058	.455	72.378	.000

## Discussion

This study is valued in that the design was a longitudinal approach and allowed to detect SDLRS and GCS progress over the school years from the time of admission. With the differences observed between the three years, the learning environment can be analyzed. One of the primary findings of the current study was that there were significant differences in the SDLRS scores during the three years after admission into the medical school. The SDLRS scores increased mostly one year after admission, and remained approximately the same for the two subsequent years. These results are different from previous studies. Medical students at Dalhousie University indicated no difference in SDLRS scores when measured longitudinally over a one-year period after making changes to the curriculum [26]; on the other hand, there was a significant decrease at the end of the first year after admission [14]. A study conducted on medical students at the University of Toronto Family of Medicine, which administered three instruments measuring SDL (e.g. SDLRS, Ryan’s ability, and importance scores), indicated that a decrease of Ryan’s instrument scores showed a decrease with more training [20]. Interestingly, the longitudinal study on nursing students who took a problem-based learning program showed an increase in SDLRS scores with school training, which implies that several factors play a role in promoting SDL readiness such as curriculum delivery strategies [26]. Based on previous research and the results of the current study, we can conclude that the learning environment is influential in fostering SDL among medical students. As has been mentioned before, our medical school changed the grading system from a multi-tiered norm-referenced grading system (e.g. A, B, C, F) to a two-tiered criterion-referenced grading system (e.g. pass, non-pass). In addition, several courses consisting of small group activities were developed to give students opportunities to engage in collaborative learning through interactions with their classmates. Although studies reported that there is no significant difference or promotes noncognitive skills in SDL after major changes to the curriculum [2, 27], we presumed that these changes may play a significant role in the increase of SDLRS scores. Specifically, scores for the SDLRS subdomains of ‘openness for learning’ and ‘basic learning function and independence’ significantly increased in the first year. This is not surprising because students had to learn a substantial body of medical knowledge, which may have led to the improvement of their learning strategies and capacity to learn. Interestingly, scores for ‘love of learning’ increased continuously over the three years. It was a positive outcome that group activities, small groups lectures, and a new grading system adopted at Yonsei University College of Medicine in 2014 complemented students’ desire for learning. The study found a high score for desire for learning in SDLRS that was explained by a hybrid curriculum. This included teaching, learning, and assessment strategies, which contributed to create a desire for learning [3]. Additionally, it is noteworthy that ‘self-perception’ decreased for learners in their third year. Perhaps students feel overwhelmed by the excessive amount of medical knowledge during medical training, which in turn influenced self-perception negatively.

Another aspect that should be considered is group cohesion. With respect to GCS scores for assessing group cohesion, the current results show that scores significantly increased as medical school training progressed. In addition, significant positive relationships were found between SDLRS and GCS scores; the higher the GCS scores, the higher the SDLRS scores. Our findings support an earlier study [28] that SDL does not only signify autonomy, but also is a concept that involves interactions or coherence with colleagues to exchange information. Additionally, GCS scores explained the variance of SDLRS partially. GCS consisted of two constructs: engagement and cohesion. According to Wongpakaran et al. [24], cohesion and engagement fall under the same umbrella but have different functions: one assesses affective cohesion (e.g. feelings of trust), while the other assesses behavioral cohesion (e.g. participation). Attention must be paid to the fact that learning behaviors such as SDL or GCS may vary widely in a new and unfamiliar context, [29, 30]. The importance of context in motivating students to become a self-directed learner and enhance group coherence must be stressed [31]. Based on the results of the current study, medical educators may design curriculum and/or a training program that fosters both affective and behavioral cohesiveness in groups. For example, small team-based projects for one semester or one year could be a requirement for all students for pre-clinical and clinical years, which might, in turn, contribute to the improvement of students’ SDL.

This study was conducted in a single institution, limiting the generalizability of its results to other medical schools. In addition, although participants were told that the identifying information was coded as anonymous and could not be assessed by researchers at all, there is the possibility of social desirability having influenced their answers. For future research, SDLRS scores for the graduation year could be also be added to the subsequent analysis, so that the overall pattern of SDLRS for the four years of medical school can be determined. Further explorations can be conducted on whether group cohesion and SDLRS act as good predictors of academic performance. Although it is vital to medical education, many questions regarding the relationship between SDL and collaboration with peers or colleagues in medical education contexts remain unanswered.

## Conclusions

This study investigated medical students’ SDLRS and GCS after admission into a medical school. It revealed significant improvements in students’ SDL readiness and group cohesion as medical school training progressed. Group cohesiveness appears to be a significant factor in fostering SDLRS. Cohesion with colleagues and SDL are important axes for successful completion of medical school. Medical schools should develop various curriculum activities that enhance group cohesion among medical students, which would in turn promote SDL.

## Data Availability

The datasets used during the current study are available from the corresponding author on reasonable request.

## Abbreviations

GCS: group cohesiveness scale
SDL: self-directed learning
SDLRS: self-directed learning readiness scale
